# P-628. Real-world impact of pneumococcal conjugate vaccines on vaccine serotypes and cross-reacting non-vaccine serotypes

**DOI:** 10.1093/ofid/ofae631.826

**Published:** 2025-01-29

**Authors:** Kevin Apodaca, Lindsay Grant, Johnna Perdrizet, Derek Daigle, Gabriel Mircus

**Affiliations:** Pfizer Inc., New York, New York; Pfizer Inc., New York, New York; Pfizer Inc, New York , New York; Pfizer, Inc, New York, New York; Pfizer, Inc, New York, New York

## Abstract

**Background:**

Cross-reactivity of vaccine-induced antibodies with some non-vaccine serotypes has been demonstrated in clinical trials and serological studies, but there is limited longitudinal data on the impact of pneumococcal conjugate vaccines (PCVs) on cross-reactive serotypes after implementation in infant immunization programs. This study describes the impact of PCVs on pneumococcal disease cases due to potential cross-reactive serotypes.

**Table 1.**
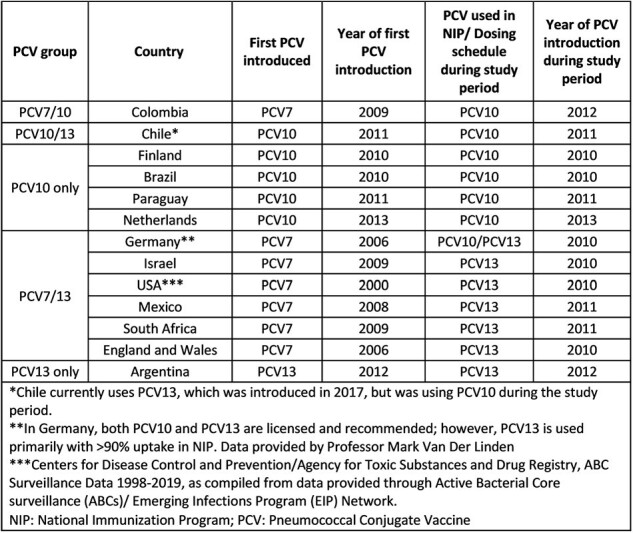
Countries included in analysis

**Methods:**

We identified 13 countries with serotyped invasive pneumococcal disease (IPD) surveillance data that had introduced PCV10 or PCV13 (Table 1). Analyses were restricted to IPD cases due to serotypes included in PCV10/13 (VTs: 6B, 9V, 18C, 19F, 23F, 7F; PCV13 only VTs: 6A, 19A) and to non-vaccine serotypes (NVTs: 6C, 7A, 7B, 7C, 9N, 18A, 18B, 18F, 23A, 23B) that could be immunologically related to VTs in children < 5 years. For each country, we compared case counts 1 year before and the latest year of data available (2018/19) after PCV introduction and calculated relative percent change.

**Results:**

Decreases in VT IPD cases were observed across all countries after PCV introduction, while some NVT IPD cases had no change or increased. VT cases declined by 59-100%. Serotype 19A cases declined by 34-100% (0-26 cases in 2018/19) in PCV13 countries but increased by 63-1460% (13-78 cases in 2018/19) in PCV10 countries. NVT 6C cases had little to no change (0-6 cases in 2018/19) in PCV13 countries but increased by 100-1200% (1-12 cases in 2018/19) in PCV10 countries. In PCV13 countries, 7B, 7C, and 9N cases did not change (0-6 cases in 2018/19), while 23A and 23B increased moderately by 200-900% (2-16 cases in 2018/19). NVT 7B, 7C, 9N, 23A, and 23B had no data available for PCV10 countries, while NVT 7A, 18A, 18B, and 18F did not have sufficient cases in any PCV setting to include in the analysis.

**Conclusion:**

The inclusion of VT 19A in PCV13 and not in PCV10 may explain the significant increase of NVT 19A cases in PCV10 countries. Conversely, the minimal change of NVT 6C cases in PCV13 countries but large increases in PCV10 countries suggest cross-protection of serotype 6C by PCV13, likely due to inclusion of serotype 6A. For other NVTs, cross-protection was not identified based on observing no change or increasing cases or could not be evaluated due to low case counts.

**Disclosures:**

**Kevin Apodaca, MPH**, Pfizer, Inc: Employee of company|Pfizer, Inc: Stocks/Bonds (Private Company) **Lindsay Grant, PhD, MPH**, Pfizer, Inc: Employee of company|Pfizer, Inc: Stocks/Bonds (Private Company) **Johnna Perdrizet, MPH**, Pfizer, Inc: Employee of company|Pfizer, Inc: Stocks/Bonds (Private Company) **Derek Daigle, PhD**, Pfizer, Inc: Employee of company|Pfizer, Inc: Stocks/Bonds (Private Company) **Gabriel Mircus, PhD**, Pfizer, Inc: Employee of company|Pfizer, Inc: Stocks/Bonds (Private Company)

